# Physicochemical Properties and Phytochemical Composition of ‘French’ Plums at Different Maturities

**DOI:** 10.3390/foods15101766

**Published:** 2026-05-17

**Authors:** Daiyi Zhao, Kaiyue Bi, Dongsheng Niu, Xuewen Li, Feng Li

**Affiliations:** College of Food Science and Pharmacy, Xinjiang Agricultural University, Urumqi 830052, China; 18997873679@163.com (D.Z.); 18160562890@163.com (K.B.); 15124789941@163.com (D.N.); xjndsp@sina.com (X.L.)

**Keywords:** ‘French’ plums, maturity, quality indicators

## Abstract

Plums are primarily sold fresh, but post-harvest softening and rotting can cause significant economic losses. Understanding quality changes across different maturity stages is crucial for meeting consumer demand for high-quality fruit. This study systematically analyzed the dynamic changes in the physicochemical properties, phenolic content, and cellular structure of ‘French’ plums during six growth and development stages (D1–D6), and comprehensively evaluated fruit quality using correlation analysis and principal component analysis (PCA). The results showed that firmness declined considerably with maturity, whereas the soluble solids content (SSC) increased and titratable acidity (TA) decreased. The peel color progressed from green to a purplish-red. The levels of sugars, such as glucose and fructose, increased, whereas those of major organic acids decreased. Phenolic content varied with developmental stage, with catechin and epicatechin peaking at the D3 stage (pre-color green stage), demonstrating exceptional antioxidant potential. At the D5 stage (purple stage), the fruit exhibits an ideal balance of sweetness, acidity and moderate firmness. Although at the D6 stage (full purple ripe stage), SSC reached its highest levels, fruit cell walls were compromised, vesicles ruptured, and firmness significantly decreased. At this stage, phenolic content declined, indicating that the fruit had attained full maturity. At this maturity level, the fruit should be promptly consumed or processed.

## 1. Introduction

*Prunus domestica* L. is a European plum species belonging to the Prunus genus of the Rosaceae family. Plums are characterized by their high-water content and rich nutrient profiles, including anthocyanins, vitamins, soluble dietary fiber, and polyphenols. These fruits contain abundant bioactive compounds that confer health benefits such as enhanced immunity, antioxidant properties, and anti-aging effects [1,2,3]. Plums are important in both the fresh food and processing sectors because of their distinctive flavor and nutritional richness [4]. The ‘French’ plum variety has emerged as the preferred cultivar for cultivation and industrialization in China, owing to its stable fruit characteristics and exceptional resistance to storage and transportation [5].

Fruit maturity is a core factor governing quality, metabolite turnover, cellular structural evolution, and utilization value of stone fruits. Fruits at different ripening stages exhibit significant differences in physicochemical properties, functional components, cellular ultrastructure, and biological activities, which directly determine the edible quality, storage life, and processing suitability of the fruits [6,7,8]. Early studies on the ripening characteristics of stone fruits have mostly focused on the determination of basic physicochemical indicators and the exploration of conventional storage and preservation technologies. Xi et al. [9] investigated the dynamic variations in fundamental physicochemical properties of sugar and organic acids during apricot fruit growth and ripening. Yan et al. [10] and Banin Sogvar et al. [11] explored the effects of exogenous melatonin and L-cysteine treatments on postharvest quality retention and storage preservation of plum fruit, respectively. However, insufficient attention has been devoted to the dynamic accumulation of functional components, the temporal evolution of cellular ultrastructure, and their correlation mechanism with fruit quality during the ripening process, resulting in insufficient systematicness and depth in relevant research. With the rapid advancement of modern food omics, microscopic imaging, and metabolic analysis technologies, recent studies have shifted toward multi-index integrated analysis. For instance, Xiao et al. [4] established a comprehensive flavor evaluation system for plum fruit through the systematic determination of sugars, organic acids, and phenolic components. Tang et al. [12] utilized UPLC-QTOF-MS-based metabolomics technology to characterize the phenolic profiles of Xinjiang plum fruit and clarify the correlation between functional components and fruit quality. Wu et al. [13] conducted a multi-dimensional comprehensive analysis of the physicochemical properties, functional phenolic compounds, and aroma characteristics of Xinjiang peach fruit. Nevertheless, for ‘French’ plums in Xinjiang, there remains a lack of comprehensive multi-dimensional systematic analysis covering basic physicochemical properties, sugar and acid profiles, functional components, and cellular structure, and no unified standards for maturity identification and graded utilization have been established to date.

Accordingly, this study used ‘French’ plums as experimental materials and systematically measured fruit color, firmness, soluble solids content (SSC), titratable acid (TA), ascorbic acid (VC), individual sugars and organic acids, phenolic compounds, and flavonoids. Additionally, the evolution of cell wall ultrastructure was observed. The variation patterns of these indicators with fruit maturity and their intrinsic correlations were clarified, and a maturity evaluation and graded utilization model was proposed. This study aims to provide theoretical support for the precise harvesting and efficient utilization of ‘French’ plums in Xinjiang.

## 2. Materials and Methods

### 2.1. Plant Materials and Reagents

The experimental material was ‘French’ plum, which was harvested from Jiashi County, Kashgar Prefecture, Xinjiang. The full bloom date of ‘French’ plum was 15 April 2023. Fruit samples were collected at 55, 70, 85, 100, 115, and 130 days after full bloom (DAFB) in 2023, with a fixed sampling period of 15-day intervals. A total of six developmental stages were established and marked as D1–D6, which were defined as follows: D1, green fruit stage; D2, late green fruit stage; D3, pre-color green stage; D4, green-yellow color transition stage; D5, purple stage; D6, full purple ripe stage. For each sampling stage, 4 kg of fruit with uniform size, free of diseases, insect pests, and mechanical damage, was harvested. All samples were transported to the laboratory within 24 h and transported under cold conditions.

The chemical reagents used in this study, including sodium hydroxide (99%), phenolphthalein (98%), oxalic acid (98%), hydrochloric acid (98%), absolute ethanol (99%), and sodium carbonate (99.5%), were procured from Zhiyuan Chemical Reagent Co., Ltd. (Tianjin, China). Ascorbic acid was obtained from Beilian Fine Chemicals Development Co., Ltd. (Tianjin, China), 2,6-dichlorophenolindophenol from Yuanye Bio-Technology Co., Ltd. (Shanghai, China), and methanol was obtained from Xinbotte Chemical Co., Ltd. (Tianjin, China). Folin–Ciocalteu reagent from Haibiao Technology Co., Ltd. (Xiamen, China), and gallic acid (98%) from Fuchen Chemical Reagent Factory (Tianjin, China). All reagents used were of analytical grade.

### 2.2. Experimental Methods

#### 2.2.1. Quality Evaluation

Fifteen fruits per developmental stage were randomly selected, and their transverse and longitudinal diameters, single fruit weight, firmness, and color were measured. Afterward, the fruits were immediately pitted, homogenized, and ground to prepare a homogenate. One portion of this homogenate was immediately filtered through four layers of gauze to obtain clear juice for the determination of SSC, TA and VC. The other portion of the homogenate was stored at −20 °C for the subsequent determination of reducing sugars, organic acids, and polyphenols.

To determine the peel color value, three points at the equatorial section of the fruit were selected for measurements. The color parameters L*, a*, b* of the plum peel were measured using an NH310 color difference meter (ThreeNH, Shenzhen, China). The total color difference (∆E) between each ripening stage and the initial stage (D1) was calculated as follows: $∆E=\sqrt{{{(L}^{*}-{L_{0}}^{*})}^{2}+{\left(a^{*}-{a_{0}}^{*}\right)}^{2}+{\left(b^{*}-{b_{0}}^{*}\right)}^{2}}$, where L_0_*, a_0_*, and b_0_* are the color parameters of the plum fruits at the D1 stage. The longitudinal and transverse diameters of the fruits during development were measured using Vernier calipers (mm), and the mass of a single fruit was determined using a BSA233S electronic balance (Sartorius, Beijing, China). The fruit shape index was calculated as the ratio of the longitudinal diameter to the transverse diameter of the fruit. A GY-4 fruit firmness tester with a probe diameter of 3 mm (Top Cloud-Agri, Hangzhou, China) was used. Three equidistant positions along the equatorial part of the fruit were selected, and the skin of the plum was peeled off before the measurement. The results were expressed in Newton (N).

For determination of SSC, The SSC of plum clear juice was determined using a PAL-1 digital handheld refractometer (Atago, Tokyo, Japan). Results were expressed as percentage (%) [3]. The TA was determined using acid-base titration and calculated using a malic acid conversion factor of 0.067 g/mmol. The 2,6-dichlorophenolindophenol titration method [14] was used to determine the VC content, which was expressed as mg/100 g.

#### 2.2.2. Sugars and Organic Acids

Soluble sugars and organic acids were identified and quantified using an established procedure [15,16] with slight adjustments. Detailed protocols are provided in Supplementary Methods S1.

#### 2.2.3. Determination of Polyphenolic Compounds

High-performance liquid chromatography was used to determine the polyphenolic compounds present [17,18,19,20]. A fruit sample (100 mg) was placed in a centrifuge tube, and 0.5 mL of 80% methanol aqueous solution (containing 0.2% VC) was added. The mixture was vortexed and ultrasonicated at room temperature for 30 min. Then, the mixture was centrifuged at 12,000 rpm for 10 min, and the supernatant was collected. The extraction was repeated twice, and the supernatants from the two extractions were combined and mixed thoroughly. The samples were analyzed using an ultra-high-performance liquid chromatography system (Vanquish, UPLC, Thermo Fisher Scientific, Waltham, MA, USA) coupled with a high-resolution mass spectrometer (Q Exactive, Thermo Fisher Scientific, Waltham, MA, USA). The mobile phases were as follows: Phase A was ultrapure water (containing 0.1% formic acid), and Phase B was acetonitrile (containing 0.1% formic acid). The flow rate was 0.3 mL/min, the column temperature was maintained at 40 °C, and the injection volume was 2 µL. The elution gradient was as follows: 0 min, A Phase/B Phase (90:10, *v*/*v*); 2 min, A Phase/B Phase (90:10, *v*/*v*); 6 min, A Phase/B Phase (40:60, *v*/*v*); 9 min, A Phase/B Phase (40:60, *v*/*v*); 9.1 min, A Phase/B Phase (90:10, *v*/*v*); and 12 min, A Phase/B Phase (90:10, *v*/*v*). Throughout the analysis, the samples were maintained at 4 °C in an autosampler. For polyphenol quantification, calibration curves with good linearity (R^2^ > 0.99) were constructed using an external standard method. These calibration curves (Figure S2) were plotted with peak area as the ordinate (*y*-axis) and the concentrations of phenolic acids and flavonoids as the abscissa (*x*-axis). All determinations were performed in triplicate, and the results are expressed as ng/100 mg fresh weight (FW).

#### 2.2.4. Transmission Electron Microscopy (TEM) Analysis

The fruit surface was rinsed with sterile normal saline to remove extraneous matter, including sediment. Sections of plum peel measuring 5  ×  5 mm were excised, fixed, and dehydrated, as described by Yao et al. [21]. Samples D1–D6 were selected for TEM to examine the cellular morphological changes (FEI Systems, Hillsboro, OR, USA). The target area was visualized using a Leica DM6 B upright microscope (Leica, Wetzlar, Germany) equipped with a Leica DFC700 T camera and LASX software (Version 3.6.0).

### 2.3. Data Analysis

All determinations were performed using three replicates and results are expressed as mean ± standard error. The experiment was performed using complete randomization of the samples. SPSS 26.0 (IBM, Chicago, IL, USA) was used for data analysis, and Duncan’s multiple range test was used to analyze the differences between maturities. Statistical significance was set at *p* < 0.05. Origin 2024 (Origin Lab Corporation, Northampton, MA, USA) was used to plot the data.

## 3. Results

### 3.1. Changes in the Physicochemical Characteristics of ‘French’ Plums Harvested at Different Maturity Stages

As the plums gradually matured, the color of the fruit changed from green to yellowish-green and finally to purplish-red. Table 1 shows the changes in color coordinates during the development of plum fruits. The L* value represents the brightness. The larger the L* value, the brighter the plum fruit peels. The L* value reached a maximum of 51.44 at the D1 stage. As the fruits gradually matured, the L* value showed a significant downward trend and suddenly dropped to 24.97 at the D5 stage, which was significantly different from the L* values measured at other maturity stages (*p* < 0.05). The b* value (yellowish-blue) exhibited a trend characterized by an initial increase, followed by a decline. Specifically, the b* value increased gradually during the D1–D4 stage, peaking at 30.85 in the D4 stage before significantly decreasing to 12.08 in the D5–D6 stage (*p* < 0.05). The a* values reflect the redness and greenness of the pericarp; thus, a higher value corresponds to a deeper red color. Concurrently, the a* value increased from −8.89 (indicating a greenish tone) at D1 to 31.7 (indicating a reddish-purple tone) at D6. The a* value transitioned from negative to positive between the D4 and D5 stages, marking a critical color transition from the green ripening stage to the mid-ripening stage. In addition to peel color, fruit shape and size also showed regular variations during fruit development. The transverse diameter of plum fruits increased rapidly from D2 to D4 and then gradually increased (Table 1). The longitudinal diameter of the fruits increased from 30.85 to 40.71 mm from the D1 to D5 stages and then entered a slow growth period from the D5 to D6 stages. The transverse growth rate of plum fruits was higher than the vertical growth rate from D1 to D4, indicating that the transverse growth of the fruits was relatively fast during this stage, and the fruits rapidly thickened. The longitudinal growth trend of the fruits increased from D4 to D5, and the fruits became longer. The longitudinal diameter of the plum was always larger than the transverse diameter during the entire developmental process, which is consistent with the oval shape of the fruits. The single-fruit mass of the plum was similar to their longitudinal diameter. The single-fruit mass increased rapidly from 15.48 to 33.24 g as the fruit grew rapidly during this stage. The fruit shape index, a commercial quality indicator for plums, was 1.18 at the D6 stage, the lowest value during the entire growth period. Therefore, at this stage, the fruit was plump, and the shape was almost round.

**Figure 1:**
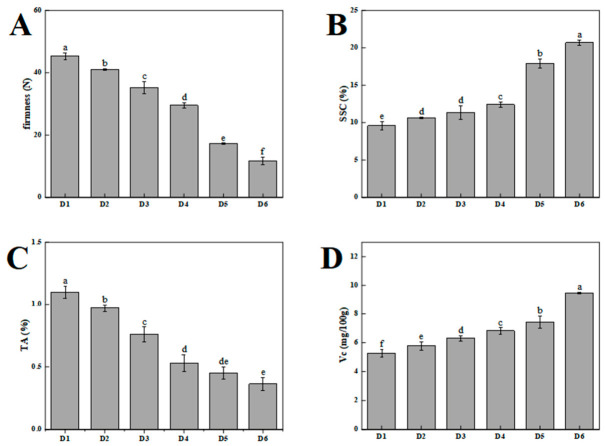
Changes in firmness (**A**), soluble solids content (SSC) (**B**), titratable acid (TA) (**C**), and ascorbic acid (VC) (**D**) of ‘French’ plums harvested at different maturity stages. Different letters indicate significant differences (*p* < 0.05) between maturity stages.

Figure 1 illustrates the changes in the quality index of plums during growth and development. As the fruit ripened, its firmness declined from 45.52 to 11.49 N, with a marked decrease post-D5 stage (*p* < 0.05) (Figure 1A). The SSC increased steadily from D1 to D4 and reached a maximum value (20.7%) at D6 (Figure 1B). Concurrently, TA decreased by 29.33% between D2 and D3, with the highest TA at 1.09% on D1 and lowest at 0.35% on D6 (Figure 1C). VC content increased from 5.26 mg/100 g to 9.48 mg/100 g, with significant (*p* < 0.05) differences between maturity stages (Figure 1D).

### 3.2. Changes in Sugar and Organic Acid Contents of ‘French’ Plums Harvested at Different Maturity Stages

Glucose, sucrose, and fructose were identified in plum fruits, with glucose exhibiting the highest concentration (Table 2). Glucose levels varied from 453.24 to 703.74 mg/100 g, increasing by 55.3% from D1 to D6. Sucrose and fructose levels were relatively low, ranging from 69.92 to 312.32 mg/100 g and 46.91 to 167.84 mg/100 g, respectively. The sucrose content in D6 fruits was 3.47 times higher than that in D1 fruits, whereas the fructose content was 2.57 times higher in D6 fruits than in D1 fruits (*p* < 0.05). Table 2 lists the five organic acids present in plum fruits: malic, quinic, shikimic, citric, and fumaric acids. The malic acid content decreased from 40.34 mg/100 g (D1) to 20.02 mg/100 g (D6) as the fruits matured. The quinic acid content exhibited an increasing pattern from 35.44 mg/100 g (D1) to 178.88 mg/100 g (D6). The levels of shikimic acid and citric acid displayed a notable trend of initial increase followed by a decrease with maturity, peaking at 3.43 mg/100 g and 1.32 mg/100 g during the D3 and D4 stages, respectively. Conversely, the fumaric acid content increased from 0.09 mg/100 g at the D1 stage to 1.39 mg/100 g at the D6 stage.

### 3.3. Changes in Phenolic Content of ‘French’ Plums Harvested at Different Maturity Stages

Table 3 shows the changes in phenolic compound content during ‘French’ plum fruit development. A total of 24 phenolic compounds were identified, including 11 flavonoids and 13 phenolic acids. Quercetin rutinoside (rutin), catechin, and epicatechin were the most abundant flavonoids in plum pulp. As shown in Table 3, rutin content decreased with increasing maturity, reaching its peak at the D1 stage (4407.2 ng/100 mg) before declining to its lowest level at the D5 stage (1969.2 ng/100 mg). The catechin and epicatechin contents exhibited a pattern of increase followed by a decrease with maturity, peaking at the D3 stage with values of 9095.93 ng/100 mg and 852.54 ng/100 mg, respectively. Additionally, the highest concentrations of kaempferol-3-O-glucoside (18.92 ng/100 mg), dihydroquercetin (22.66 ng/100 mg), and quercetin-3-β-D-glucoside (385 ng/100 mg) were observed at D1, D3, and D4, respectively. Low levels of luteolin, dihydromyricetin, quercetin, naringenin chalcone, and luteoloside were also detected, with slight variations in their content throughout the ripening process. The total flavonoid content of plum fruits ranged from 7504.58 ng/100 mg to 12,501.75 ng/100 mg, reflecting trends similar to those of catechin and epicatechin. The total flavonoid content of plum fruits initially increased, followed by a decrease as the fruit ripens.

Thirteen phenolic acids were identified in plum fruit, with protocatechualdehyde, caffeic acid, vanillic acid, gallic acid, and vanillin as the predominant compounds. Protocatechualdehyde was the most abundant phenolic acid, exhibiting a peak concentration of 67.21 ng/100 mg at the D2 stage. Additionally, gallic acid (7.31 ng/100 mg) and vanillic acid (7.75 ng/100 mg) were detected at the D2 and D3 stages, respectively, whereas higher concentrations of vanillin (9.44 ng/100 mg), caffeic acid (8.3 ng/100 mg), and trans-cinnamic acid (23.79 ng/100 mg) were observed at the D5 stage. Lower levels of syringaldehyde, phthalic acid, syringic acid, p-hydroxycinnamic acid, salicylic acid, trans-ferulic acid, and hydrocinnamic acid were also detected, showing relatively minor fluctuations in their content throughout the ripening process. Furthermore, the trend in total phenolic acid content paralleled that of the total flavonoid content.

### 3.4. Transmission Electron Microscopy (TEM) Analysis of ‘French’ Plum Peels

**Figure 2:**
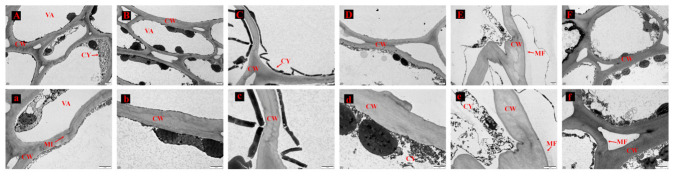
Electron microscopy of the pericarp of ‘French’ plums harvested at different maturity stages. (**A**–**F**): Cell ultrastructure of D1–D6 stage fruits (×1000), respectively; (**a**–**f**): Cell wall and matrix of D1–D6 stage fruits (×2500), respectively (ML, middle lamella; CW, cell wall; MF, microfibril; VA, vacuole; CY, cytosol).

Figure 2 illustrates the structural alterations in the cell matrix and cell wall of plum peels at various ripening stages (D1–D6). In the initial stages, the pericarp cell wall thickness was consistent, and the vesicle morphology appeared regular (Figure 2A,B). The cytoplasmic solubles were uniformly distributed and filled the cells, whereas the middle lamella maintained a tight connection to the cell wall (Figure 2A,B). During the D3–D4 stages, the pericarp cell wall exhibited slight thinning, accompanied by an expansion of the vesicle volume (Figure 2C,D). The distribution of cytoplasmic solubles further diminished, with some areas exhibiting contraction, and the connectivity of the middle layer to the cell wall weakened (Figure 2c,d). At the D3–D4 stage, the pericarp cell wall continued to thin slightly, and the vesicle volume expanded marginally (Figure 2C,D). The distribution of the cytoplasmic lysate narrowed, with contractions observed in certain areas, leading to a further reduction in the connectivity of the middle layer to the cell wall (Figure 2C,D). Ultimately, the cytoplasmic lysate nearly vanished, the middle layer largely disappeared, the cell gaps increased, and the cell wall released a significant number of microfilaments (Figure 2e,f).

### 3.5. Correlation Analysis of Quality Indicators During the Growth and Development of ‘French’ Plum Fruit

**Figure 3:**
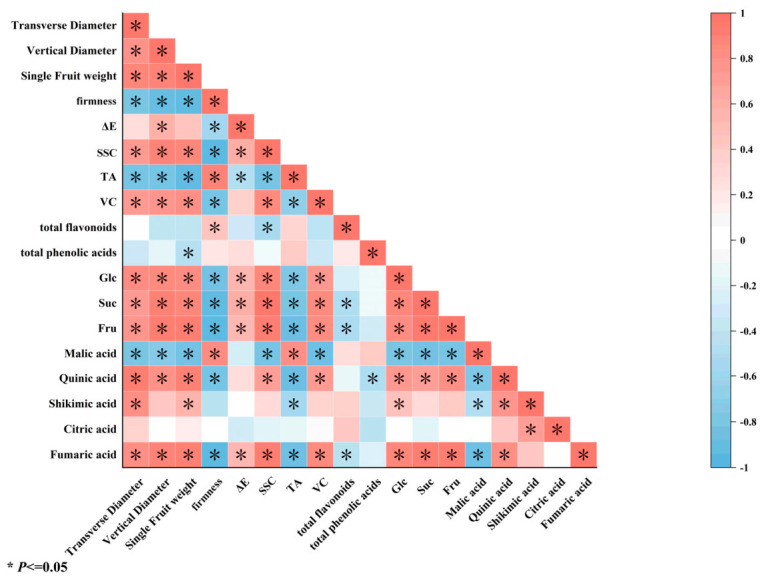
Pearson correlation heatmap. The color gradient (from blue to red) represents the magnitude of the Pearson correlation coefficient: blue indicates a negative correlation, while red indicates a positive correlation; the darker the color, the larger the absolute value of the correlation coefficient, and the superscript “*” indicates that the correlation is significant at *p* < 0.05.

Figure 3 presents a Pearson correlation heatmap illustrating the relationships between various quality indicators during plum fruit development. The fruit morphology indicators, namely transverse diameter, longitudinal diameter, and single fruit weight, exhibited a strong positive correlation with each other. Additionally, they showed a significant negative correlation with firmness and significant positive correlations with SSC, glucose, sucrose, and fructose. Furthermore, these morphological indicators were positively correlated with TA and color difference ΔE. Firmness was positively correlated with SSC but negatively correlated with SSC and individual sugars (glucose, sucrose, and fructose). The individual sugars were positively correlated with each other and with the SSC. TA showed a significant positive correlation with malic acid, which was negatively correlated with morphological indices. Quinic acid exhibited a positive correlation with morphological indices, whereas citric acid showed no significant correlation. VC was significantly and positively correlated with the morphological indices, sugar indices, and SSC. Moreover, total flavonoids and total phenolic acids were positively correlated with SSC, and total flavonoids were positively correlated with phenolic acids. The significant correlations between these indices indicate that the characteristics of plum fruits do not vary independently but form various correlation clusters. To simplify the complex interrelationships between multiple indices and to refine the core factors influencing fruit quality, further principal component analysis may be warranted.

### 3.6. Principal Component Analysis (PCA)

**Figure 4:**
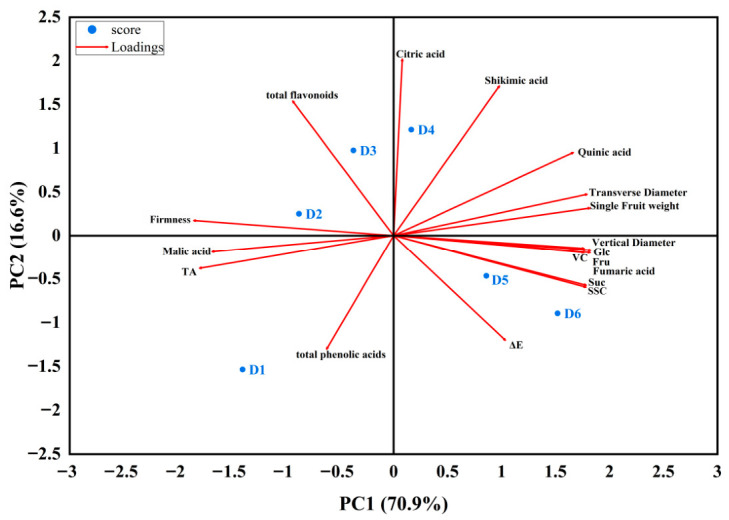
Principal component analysis (PCA) for ‘French’ plums harvested at different maturity stages.

Principal component analysis was employed to objectively assess the impact of various quality indicators on plum quality. Principal components 1 (PC1) and 2 (PC2) explained 70.9% and 16.6% of the total variance, respectively, contributing to a cumulative variance of 87.5%. This suggests their adequacy in capturing primary variance in the dataset. In Figure 4, early maturity stage fruits (D1, D2, and D3) were on the left side of PC1, whereas more mature fruits (D4, D5, and D6) were on the right side of PC1. Fruits in the early maturity stage were distinguished by their firmness, malic acid content, TA, total flavonoid content, and total phenolic content. Conversely, fruit morphology (transverse diameter, longitudinal diameter, and single fruit weight), VC content, SSC, glucose, fructose, and sucrose were the primary distinguishing factors for riper fruit. For PC2, the strong positive eigenvectors were associated with total flavonoids and citric acid, which were notably higher in fruits at stages D3 and D4. Furthermore, the eigenvectors for firmness, malic acid, and TA pointing in the negative direction of PC1 contrasted with the vectors for fruit morphology, SSC, glucose, fructose, sucrose, and vitamin C in the positive direction of PC1, indicating a significant negative correlation between these quality characteristics.

## 4. Discussion

Fruit appearance and morphology are core quality indicators that directly reflect maturity, commercial value and consumer preference. In this study, the surface color of plums harvested at different maturity stages changed from green to purplish-red, with a decrease in the L* (dark to light) value and an increase in the a* (green to red) value. Barrett et al. [22] stated that the main pigments determining the color quality of the fruits are fat-soluble chlorophylls (green), carotenoids (yellow, orange, and red), water-soluble anthocyanins (red, blue) and flavonoids (yellow). Dai et al. [23] reported that the maturity of peach fruits showed a significant negative correlation with chlorophyll content and a positive correlation with anthocyanin content. In this study, the increase in the a* value and the decrease in the L* value in plums might be related to the degradation of chlorophylls and the synthesis and accumulation of anthocyanins. Beyond the variation in peel color, dynamic changes in fruit morphology and size also represent important physiological characteristics of plum fruit maturation. From the perspective of growth and development dynamics, the developmental period of plum fruit was approximately 100 days. Fruit weight, transverse diameter, and longitudinal diameter all showed a continuous increasing trend: fruit weight exhibited the largest increase (27.48%) from D3 to D4, representing the rapid accumulation period of fresh weight; rapid growth of transverse diameter mainly occurred at D2–D3 (34.19%), while longitudinal diameter increased sharply at D5 (14.90%). It was previously shown that 70–100 d after flowering and fruit setting is the critical period for rapid fruit expansion, which constitutes the key window determining final fruit size and marketable fruit rate. The fruit shape index can directly characterize fruit morphology: an index close to 1.0 indicates round fruit, and greater than 1.0 indicates elliptical fruit [24]. In this study, the fruit shape index of plums gradually decreased with ripening. Fruits at D1 were relatively long-elliptical, while those at D6 exhibited the fullest and roundest shape, indicating that the lateral growth rate gradually increased as the fruit ripened, and the fruit morphology was more consistent with the standard of high-quality commercial fruits [25].

SSC and TA are the main attributes that determine the flavor quality and are also considered key factors in fruit ripening [26]. As an important component of fruit flavor, sugars are involved in gene expression during fruit growth and plant development [27,28]. Fruits at advanced ripeness stages exhibit higher SSC and lower TA. These fruits also contain elevated levels of glucose and sucrose. Previous research has established a positive correlation between ripeness and sugar content in cherry [29] and apricots [30], which is consistent with the findings from the current study for plums. Consequently, riper fruit (D6) are sweeter and may appeal more to consumers who prefer sweet flavors. Furthermore, the organic acid content in these fruits was significantly reduced, with the malic acid concentration at the D6 stage being only half of that observed at the D1 stage, consistent with the observations of Li et al. [31]. Previous studies have shown that as fruit ripens, the content of individual organic acids decreases due to increased respiration, conversion of acids into other compounds, and reduced ability to synthesize acids [32]. Therefore, the decrease in malic acid content during plum fruit ripening could be due to the depletion of malic acid by respiratory metabolism. Quinic acid content continued to increase with ripening, which may be related to the enhanced enzymatic activity of the shikimic acid pathway during fruit ripening, which serves as a secondary metabolite precursor and may be involved in the regulation of phenolic compound synthesis. However, malic acid, citric acid, and fumaric acid exhibited distinct dynamic variation trends, which could be attributed to their different physiological functions and independent metabolic regulation mechanisms in the tricarboxylic acid (TCA) cycle [33].

Fruit firmness is an important indicator of fruit quality and can intuitively reflect the degree of maturity and the effect of post-harvest storage. Fruit firmness gradually decreased during plum fruit development. Similarly, Y. Jiang et al. [34] found that changes in cell structure during fruit ripening were associated with changes in firmness. Fully ripe (D6) fruits exhibited gradual loosening of cell membrane content and cell wall connections, thinning of the cell wall, and an increase in cell gaps, indicating that they were fully ripe. Deng et al. [35] noted that the softening phenomenon in mango fruit could be attributed to the degradation of the cell wall, in which a variety of hydrolytic enzymes, such as pectinase, polygalacturonase, and cellulase, work together to break down the pectin components. Therefore, during fruit ripening, the significant increase in pectinase activity leads to the rapid degradation of the pectin fraction, which may be the main reason for the gradual softening of plum fruit. In addition, related studies have shown that highly mature apricot fruits with lax cellular structures and degradation of pectin in the cell wall are prone to water loss and shriveling [34]. Based on these results, to preserve the quality of D6-stage fruit and avoid quality deterioration, rapid dehydration for dried fruit production or heating and concentration for jam manufacturing represent suitable post-harvest utilization strategies.

Phenolic compounds are secondary metabolites widely distributed in plants [36]. They possess antioxidant, anticancer, antibacterial, and anti-inflammatory activities [3]. Consequently, higher phenolic content is associated with health benefits. The concentrations of catechin and epicatechin increased significantly during the D1–D3 stages. The D3 stage exhibited the highest levels of catechins, which are prevalent polyphenolic compounds in plants that contribute to various bioactive functions and physiological and health-related effects [37]. Hence, fruits at the D3 stage may exhibit elevated biological activity. Previous research has demonstrated notable variations in enzyme activity among fruits at distinct maturity stages, influencing secondary metabolite content [38]. The activation of synthase activity for catechin and epicatechin in D3 stage plums, which resulted in an increase in the concentration of secondary metabolites, may account for the elevated levels of these two phenolic compounds. Furthermore, results from this study indicated that significant alterations in fruit cellular structure occurred during the D5–D6 stage, coinciding with a notable decrease in phenolic content. Consequently, the destruction of cellular tissue, coupled with the release of vesicle hydrolases during fruit ripening, is a key factor contributing to the reduction in phenolic content [39]. In addition, researchers have found that phenylalanine ammonia-lyase (PAL) activity decreases during the late ripening stage of pear fruit, resulting in a decrease in phenolic content [40]. The significant decrease in phenolic content at the D5–D6 stage may be due to the weakening of synthetase activity and the enhancement of degradative enzyme activity. Based on the results discussed above, due to their high phenolic content, plums at the D3 maturity stage can be used to produce nutraceuticals, food additives, and natural preservatives. A previous study found a positive correlation between maturity and VC content in the fruits of Japanese plum varieties [41], which is highly consistent with the results from the present study. In our study, the trend of single antioxidant content differed from that of phenolic content. Phenolic and VC accumulation patterns during fruit ripening are significantly different, with phenolic compounds contributing more to antioxidant activity [42]. We hypothesized that the metabolic regulation pathways of phenolics and VC during plum fruit development are relatively independent and that they may have synergistic or complementary roles in antioxidant functions.

The principal component analysis (PCA) results from this study clearly reflected the core biological changes of ‘French’ plums during ripening. The first principal component (PC1), with a contribution rate of 70.9%, distinctly illustrated the typical transition process of plum fruit: the early developmental stage was dominated by firmness maintenance and organic acid accumulation, while the late ripening stage was characterized by sugar accumulation and fruit expansion. The second principal component (PC2) with a contribution rate of 16.6% corresponded to the pre-veraison stage D3, wherein the synthesis of flavonoids reached its peak value. The above findings can provide an important practical reference for plum production. This study screened key indicators suitable for evaluating the ripening degree of plums. Firmness, TA, and total flavonoid content can be used to identify the early developmental stage, while SSC, fruit size, and sugar components are applicable to ripeness classification. The results can offer a direct theoretical basis for targeted harvesting decisions for different utilization purposes.

## 5. Conclusions

This study systematically revealed the changes in physicochemical quality, phytochemical composition, and cellular ultrastructure of Xinjiang ‘French’ plums at six ripening stages. Combined with correlation analysis and principal component analysis, the core regulatory role of ripening progression in fruit quality formation was clarified. During ripening of plum fruit, firmness decreased continuously, SSC and sugar contents increased significantly, while TA declined gradually. Phenolic functional compounds accumulated in a stage-dependent manner, and the cell wall structure was markedly degraded at the full ripening stage. The results indicated that the contents of phenolic compounds and antioxidant activity reached the maximum level at stage D3 (pre-veraison green fruit stage). This stage is suitable for targeted harvesting as raw materials for functional foods, although the fresh edible quality is unsatisfactory. Stage D5 (purple-red stage) presents balanced sweet-sour taste and moderate flesh firmness, which is the optimal harvesting period for consumers who prefer sweet-sour taste, as well as for fresh fruit marketing and circulation, and can meet the quality demands of growers and the fresh food industry. Stage D6 (fully purple-red mature stage) exhibits the highest sugar content and sweetness, which is more suitable for the processing of plums, dried fruit, jam, and other products. Nevertheless, the cellular structure is damaged, and the flesh becomes overly soft at this stage, requiring immediate processing after harvest. In conclusion, growers, fresh food circulation enterprises, and fruit processing enterprises can implement precise staged harvesting management based on the differential quality characteristics of plums at different ripening stages. This strategy can promote the diversified industrial development of fresh plum sales, conventional processing, and deep processing of functional products.

## Figures and Tables

**Table 1:** Changes in the physical characteristics of ‘French’ plums harvested at different maturity stages.

	D1	D2	D3	D4	D5	D6
Surface color	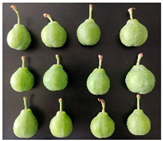	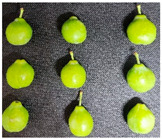	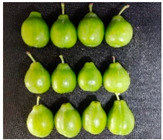	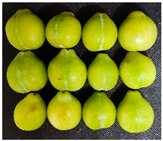	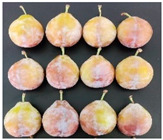	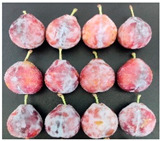
L*	51.44 ± 1.90 ^a^	50.60 ± 2.07 ^a^	46.29 ± 2.61 ^b^	41.60 ± 1.31 ^c^	24.97 ± 3.44 ^d^	24.21 ± 4.09 ^d^
b*	27.57 ± 2.73 ^b^	28.25 ± 1.05 ^b^	28.62 ± 1.57 ^ab^	30.85 ± 0.95 ^a^	19.59 ± 4.30 ^c^	12.08 ± 2.14 ^d^
a*	−8.89 ± 1.58 ^e^	−6.67 ± 1.43 ^d^	−6.38 ± 1.13 ^cd^	−4.53 ± 1.79 ^c^	15.17 ± 2.46 ^b^	31.70 ± 3.16 ^a^
Transverse Diameter (mm)	16.82 ± 0.92 ^e^	22.57 ± 0.58 ^d^	27.24 ± 0.62 ^c^	28.03 ± 0.59 ^c^	31.46 ± 0.93 ^b^	34.63 ± 0.14 ^a^
Vertical Diameter (mm)	30.85 ± 1.55 ^c^	31.26 ± 1.05 ^c^	34.92 ± 0.69 ^b^	35.43 ± 1.69 ^b^	40.71 ± 1.01 ^a^	41.38 ± 0.96 ^a^
Single Fruit weight (g)	15.48 ± 3.51 ^a^	18.41 ± 4.11 ^a^	22.34 ± 2.47 ^b^	28.48 ± 1.01 ^c^	30.62 ± 2.61 ^c^	33.24 ± 3.74 ^cd^
Fruit Shape Index	1.83 ± 0.05 ^a^	1.39 ± 0.02 ^b^	1.29 ± 0.07 ^b^	1.27 ± 0.06 ^b^	1.32 ± 0.01 ^a^	1.18 ± 0.02 ^c^

Notes: Significant differences are indicated by different superscript letters in the same row (*p* < 0.05).

**Table 2:** Changes in sugar and organic acid content of ‘French’ plums harvested at different maturity stages (mg/100 g FW).

	Sugars			Organic Acids				
	Glucose	Sucrose	Fructose	Malic Acid	Quinic Acid	Shikimic Acid	Citric Acid	Fumaric Acid
D1	453.24 ± 3.89 ^c^	69.92 ± 7.43 ^f^	46.91 ± 5.27 ^e^	40.34 ± 0.36 ^a^	35.44 ± 3.27 ^d^	0.77 ± 0.05 ^e^	0.52 ± 0.10 ^d^	0.09 ± 0.01 ^d^
D2	544.86 ± 5.22 ^b^	99.87 ± 4.35 ^e^	47.87 ± 11.58 ^e^	36.01 ± 2.77 ^b^	88.62 ± 17.20 ^c^	2.59 ± 0.13 ^d^	0.86 ± 0.12 ^c^	0.21 ± 0.04 ^d^
D3	553.23 ± 10.73 ^b^	114.64 ± 3.19 ^d^	78.67 ± 11.32 ^d^	31.82 ± 2.72 ^c^	137.99 ± 7.51 ^b^	3.43 ± 0.13 ^a^	1.08 ± 0.15 ^b^	0.52 ± 0.10 ^c^
D4	559.46 ± 7.38 ^b^	129.78 ± 8.76 ^c^	108.17 ± 8.52 ^c^	31.06 ± 0.97 ^c^	169.85 ± 6.61 ^a^	3.19 ± 0.15 ^b^	1.32 ± 0.10 ^a^	0.61 ± 0.07 ^c^
D5	677.74 ± 13.66 ^a^	250.38 ± 9.61 ^b^	146.64 ± 1.95 ^b^	30.69 ± 2.10 ^c^	165.90 ± 5.42 ^a^	2.88 ± 0.03 ^c^	0.90 ± 0.08 ^bc^	1.00 ± 0.17 ^b^
D6	703.74 ± 3.49 ^a^	312.32 ± 3.99 ^a^	167.84 ± 14.20 ^a^	20.02 ± 2.77 ^d^	178.88 ± 2.40 ^a^	2.68 ± 0.03 ^d^	0.59 ± 0.08 ^d^	1.39 ± 0.11 ^a^

Notes: Different letters within each column indicate significant differences (*p* < 0.05).

**Table 3:** Changes in phenolic compounds of ‘French’ plums harvested at different maturity stages (ng/100 mg FW).

Phenolic Profiles	D1	D2	D3	D4	D5	D6
flavonoids						
Quercetin rutinoside	4407.20 ± 192.77 ^a^	3368.26 ± 188.57 ^b^	2392.59 ± 491.64 ^cd^	2600.55 ± 438.25 ^c^	1969.20 ± 256.48 ^d^	2500.50 ± 57.27 ^cd^
Catechin	3680.38 ± 259.33 ^e^	6844.02 ± 168.55 ^b^	9095.93 ± 267.14 ^a^	5940.49 ± 175.98 ^c^	5683.84 ± 157.40 ^c^	4199.31 ± 91.84 ^d^
Epicatechin	282.33 ± 13.83 ^d^	693.20 ± 15.60 ^b^	852.54 ± 33.96 ^a^	850.55 ± 39.34 ^a^	587.69 ± 6.24 ^c^	580.61 ± 9.41 ^c^
Quercetin 3-β-D-glucoside	221.17 ± 5.00 ^b^	168.69 ± 12.20 ^c^	120.90 ± 2.61 ^e^	385.00 ± 13.12 ^a^	143.92 ± 10.87 ^d^	211.20 ± 8.69 ^b^
Kaempferol-3-O-glucoside	18.92 ± 2.66 ^a^	12.35 ± 0.96 ^b^	10.34 ± 7.62 ^bc^	6.55 ± 1.60 ^bc^	4.79 ± 1.03 ^c^	4.87 ± 2.95 ^c^
(+)-Dihydroquercetin	4.16 ± 0.71 ^b^	4.44 ± 1.36 ^b^	22.66 ± 12.93 ^a^	1.11 ± 0.74 ^b^	2.72 ± 0.86 ^b^	0.93 ± 1.28 ^b^
Luteolin	3.83 ± 2.31 ^a^	1.86 ± 0.30 ^b^	1.46 ± 0.28 ^b^	1.11 ± 0.07 ^b^	1.74 ± 0.44 ^b^	1.04 ± 0.37 ^b^
Dihydromyricetin	4.33 ± 3.49 ^a^	2.31 ± 4.00 ^b^	1.74 ± 1.97 ^c^	-	-	-
Quercetin	1.73 ± 0.03 ^a^	1.10 ± 0.55 ^bc^	1.31 ± 0.38 ^ab^	0.44 ± 0.10 ^d^	0.59 ± 0.24 ^cd^	1.07 ± 0.36 ^bc^
Naringenin chalcone	-	0.07 ± 0.13 ^c^	-	-	0.68 ± 0.29 ^b^	1.43 ± 0.60 ^a^
Luteoloside	1.45 ± 0.05 ^d^	2.47 ± 0.40 ^cd^	4.28 ± 0.95 ^b^	3.39 ± 0.14 ^bc^	8.00 ± 1.90 ^a^	3.61 ± 0.22 ^bc^
total flavonoids	8625.50 ± 402.69 ^d^	11,098.76 ± 255.23 ^b^	12,501.75 ± 707.72 ^a^	9791.18 ± 503.30 ^c^	8403.18 ± 156.01 ^d^	7504.58 ± 155.36 ^e^
phenolic acids						
Protocatechualdehyde	63.55 ± 1.58 ^a^	67.21 ± 4.76 ^a^	47.35 ± 1.42 ^b^	19.97 ± 4.29 ^d^	35.54 ± 1.68 ^c^	32.27 ± 6.82 ^c^
Vanillin	4.92 ± 0.36 ^bc^	3.83 ± 0.65 ^c^	5.54 ± 0.13 ^bc^	5.18 ± 1.39 ^bc^	9.44 ± 1.31 ^a^	6.71 ± 1.07 ^b^
Syringaldehyde	2.23 ± 0.14 ^a^	2.62 ± 0.99 ^a^	2.82 ± 1.06 ^a^	3.38 ± 0.89 ^a^	3.72 ± 1.81 ^a^	-
Phthalic acid	3.26 ± 0.28 ^bc^	3.38 ± 0.53 ^bc^	4.09 ± 0.70 ^ab^	1.94 ± 0.71 ^c^	5.27 ± 1.86 ^a^	5.61 ± 0.29 ^a^
Gallic acid	6.02 ± 0.22 ^a^	7.31 ± 1.68 ^a^	5.71 ± 1.79 ^a^	4.65 ± 1.99 ^a^	5.12 ± 2.20 ^a^	5.39 ± 2.44 ^a^
Vanillic acid	5.30 ± 0.98 ^ab^	6.57 ± 2.34 ^a^	7.75 ± 1.70 ^a^	3.84 ± 0.85 ^b^	2.58 ± 1.24 ^b^	2.75 ± 1.04 ^b^
Caffeic acid	7.27 ± 0.30 ^a^	5.60 ± 0.58 ^ab^	6.21 ± 1.31 ^a^	2.64 ± 0.83 ^b^	8.30 ± 3.76 ^a^	6.85 ± 1.34 ^a^
Syringic acid	1.14 ± 0.44 ^c^	1.61 ± 0.47 ^bc^	2.14 ± 0.49 ^ab^	1.26 ± 0.42 ^bc^	2.07 ± 0.51 ^a^	2.60 ± 0.51 ^ab^
p-Hydroxycinnamic acid	2.81 ± 0.29 ^b^	2.06 ± 0.28 ^bc^	1.58 ± 0.47 ^bc^	-	6.56 ± 3.07 ^a^	2.75 ± 0.15 ^b^
Salicylic acid	2.28 ± 0.20 ^a^	0.62 ± 0.25 ^c^	0.49 ± 0.09 ^c^	0.39 ± 0.26 ^c^	1.55 ± 0.97 ^ab^	0.85 ± 0.36 ^bc^
trans-Ferulic acid	2.39 ± 0.19 ^a^	1.34 ± 0.29 ^b^	1.24 ± 0.33 ^b^	0.98 ± 0.54 ^b^	1.73 ± 0.71 ^ab^	1.06 ± 0.65 ^b^
Hydrocinnamic acid	0.21 ± 0.21 ^a^	0.55 ± 0.39 ^a^	0.33 ± 0.29 ^a^	0.48 ± 0.12 ^a^	0.48 ± 0.25 ^a^	0.45 ± 0.18 ^a^
trans-Cinnamic acid	-	-	-	-	23.79 ± 10.89 ^a^	4.32 ± 1.49 ^b^
total phenolic acids	101.38 ± 3.03 ^ab^	102.69 ± 6.30 ^ab^	85.26 ± 3.34 ^bc^	44.71 ± 7.65 ^d^	106.14 ± 17.83 ^a^	71.61 ± 12.22 ^c^

Notes: Total phenolic acid and total flavonoid contents are expressed as the sum of the contents of the individual monomers. Different letters within each column indicate significant differences (*p* < 0.05) between maturities.

## Data Availability

The original contributions presented in this study are included in the article/supplementary material. Further inquiries can be directed to the corresponding author.

## Supplementary Materials

The following supporting information can be downloaded at: https://www.mdpi.com/article/10.3390/foods15101766/s1. Method S1: Determination of soluble sugars and organic acids; Figure S1: Calibration curves for quantification of soluble sugars and organic acids; Figure S2: Calibration curves for quantification of polyphenolic components.
