# Supplementary material for: Physicochemical Properties and Phytochemical Composition of ‘French’ Plums at Different Maturities
Source: Foods. 2026 May 17;15(10):1766. doi: 10.3390/foods15101766 (PMC13206176; doi:10.3390/foods15101766)
Supplement: Supplementary file 1 [file foods-15-01766-s001.zip › foods-4280054-supplementary.pdf]

Supplementary materials

Physicochemical Properties and Phytochemical Components of Prunes at Different Maturities

Daiyi Zhao, Kaiyue Bi, Dongsheng Niu, Xuewen Li, Feng Li,*

College of Food Science and Pharmacy, Xinjiang Agricultural University

Corresponding author
Feng Li
Tel/Fax: +86 18089209017
E-mail: lifeng@xjau.edu.cn
E-mail address for each author:
Daiyi Zhao: 18997873679@163.com
Kaiyue Bi: 18160562890@163.com
Dongsheng Niu: 15124789941@163.com
Xuewen Li: xjndsp@sina.com

Method S1:Determination of soluble sugars and organic acids

Soluble sugars were determined using HPLC following the previously reported method (Li et al., 2014). An appropriate amount (100 mg) of each sample was weighed and ground into a slurry using liquid nitrogen. Subsequently, 700 μL of 80% ethanol solution was mixed with the sample and incubated at 50 °C for 2 h. 700 μL of an aqueous solution was added for dilution. The mixture was centrifuged at 10,000 rpm for 3 min, and the supernatant was aspirated and transferred to a new tube. The chromatographic system used was a Thermo ICS 5000+ ion chromatography system (ICS 5000+, Thermo Fisher Scientific, USA), and the sugar components were analyzed and detected using an electrochemical detector. A CarboPac™ PA1 (250×4.0 mm) liquid chromatography columA: Phase B (0:100, v/v); 25 min, Phase A: Phase B (0:100, v/v); 40 min, Phase A: Phase B (95:5, v/v); and 60 min, Phase A: Phase B (95:5, v/v).

To determine the organic acids (Khan et al., 2020), 40 μL of the sample was transferred into a centrifuge tube, 1 mL of extraction solution (methanol/chloroform, 7:3, v/v) was added, mixed thoroughly, and incubated on ice for 30 min for extraction. Then, 0.6 mL of the solution was added and mixed well. The samples were centrifuged at 12,000 rpm for 10 min at 4 °C. The supernatant was then collected. The extraction was repeated twice, and the supernatants were combined for subsequent use. Ultra-high-performance liquid chromatography (UPLC, Vanquish, Thermo Scientific, USA) and high-resolution mass spectrometry (Q Exactive, Thermo Scientific, USA) were used for the analysis. Mobile phases: Phase A was ultrapure water (containing 0.1% formic acid), and Phase B was acetonitrile solution (containing 0.1% formic acid); flow rate was 0.35 mL/min; column temperature was 40 °C; injection volume was 2 μL; elution gradient: at 0 min, A Phase/B Phase (90:10, v/v), at 2 min, A Phase/B Phase (90:10, v/v), at 12 min, A Phase/B Phase (10:90, v/v), at 14 min, A Phase/B Phase (10:90, v/v), at 14.1 min, A Phase/B Phase (90:10, v/v), at 16 min, A Phase/B Phase (90:10, v/v). During the entire analysis process, the samples were placed in an autosampler at 4 °C. Sugar and organic acid standards were identified based on peak retention time and quantified based on peak area. Sugars and organic acids were measured three times, and the results are expressed in μg/ml.

Qualitative analysis of sugars and organic acids was performed by comparing their retention times with those of authentic standards. Quantitative analysis was carried out using the external standard method. Calibration curves with favorable linearity ($R^2 > 0.994$) were constructed with peak area as the ordinate (y-axis) and concentrations of sugars and organic acids as the abscissa (x-axis) (Figure S1). All measurements were conducted in triplicate, and results are expressed as mg/100 g FW.

References

[1] Li X, Wu B, Guo Q.(2014) Effects of nitric oxide on postharvest quality and soluble sugar content in papaya fruit during ripening. Journal of Food Processing and Preservation, 2014, 38(1): 591-599.

[2]Khan N, Ali S, Zandi P.(2020) Role of sugars, amino acids and organic acids in improving plant abiotic stress tolerance. Pak. J. Bot, 2020, 52(2): 355-363.

Figure S1: Calibration curves for quantification of soluble sugars and organic acids.

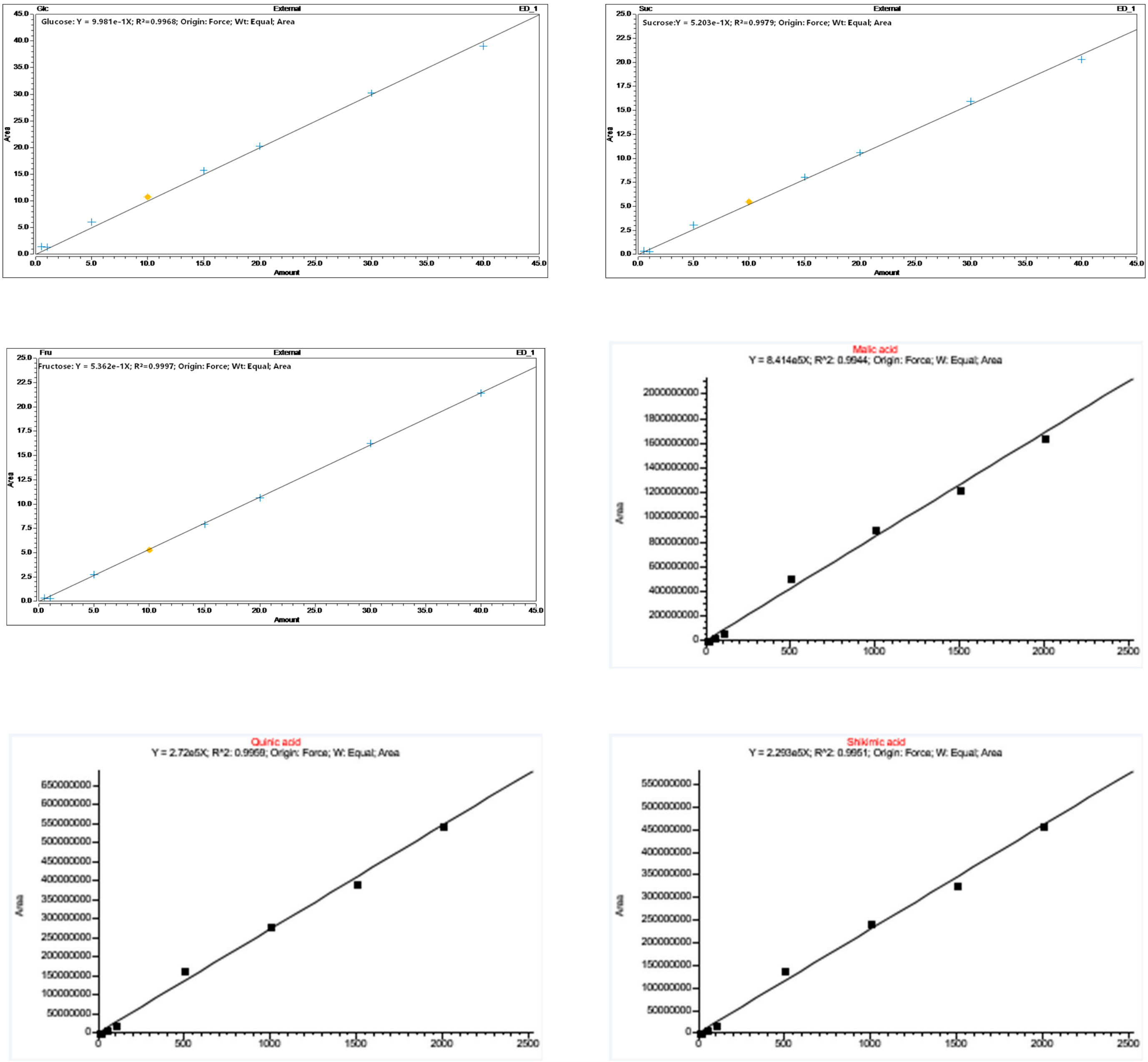

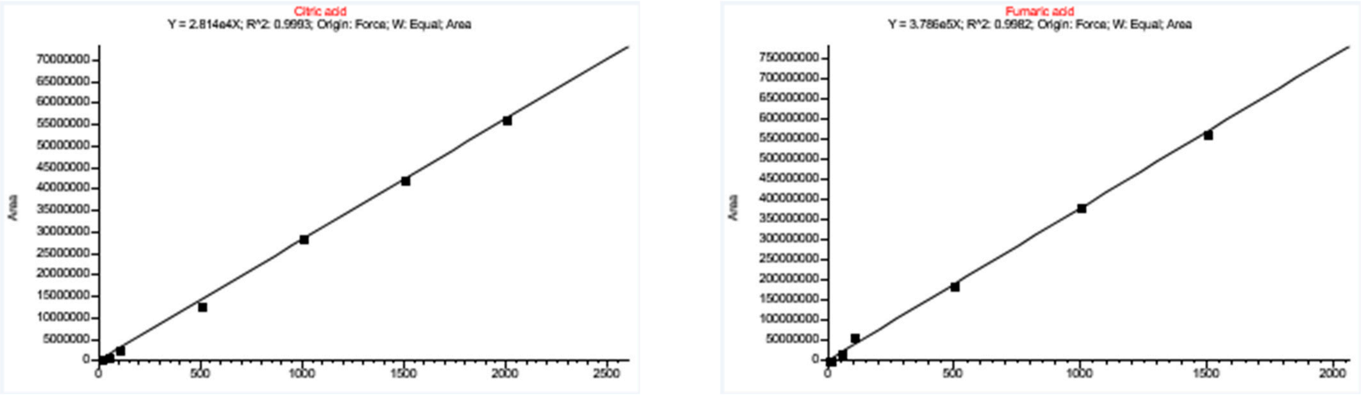

Figure S2: Calibration curves for quantification of polyphenolic components.

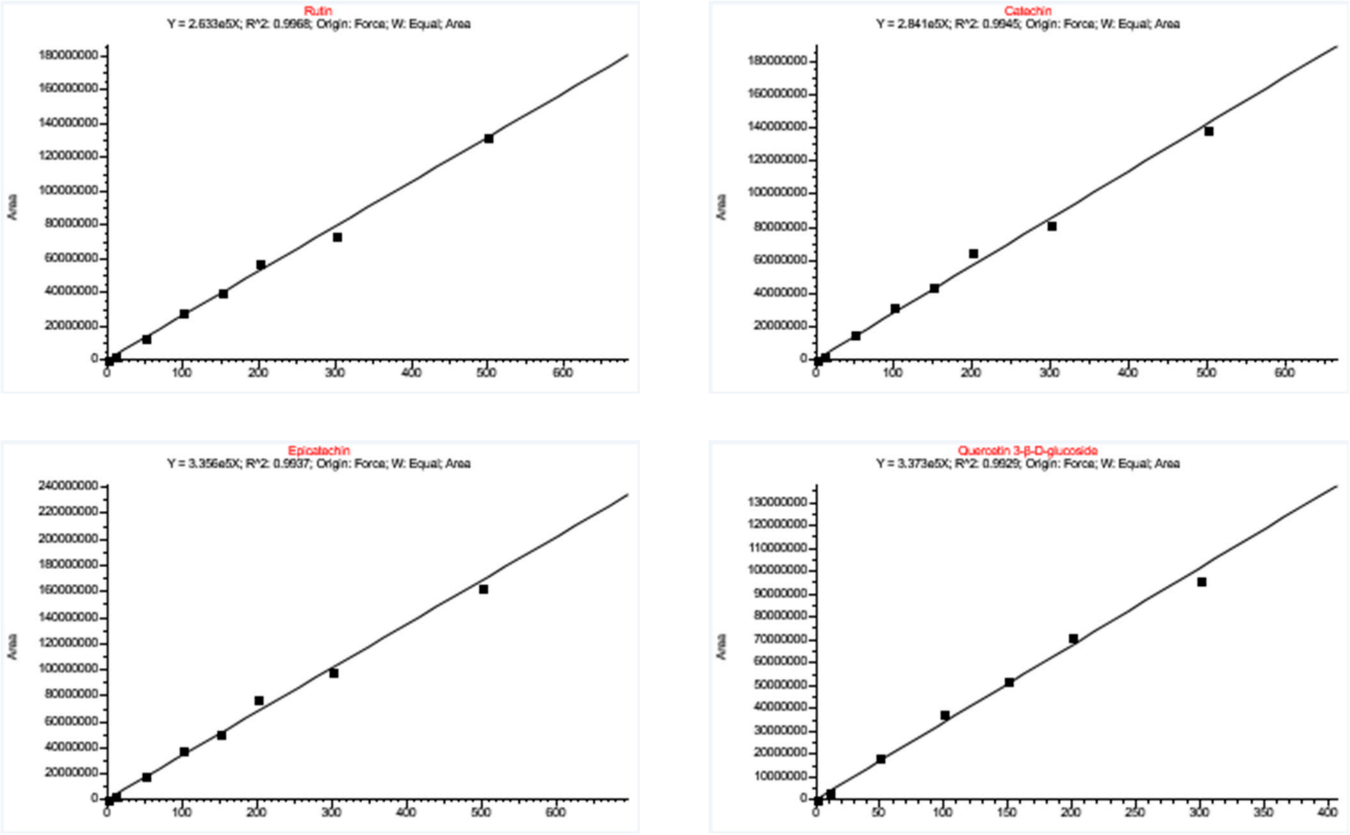

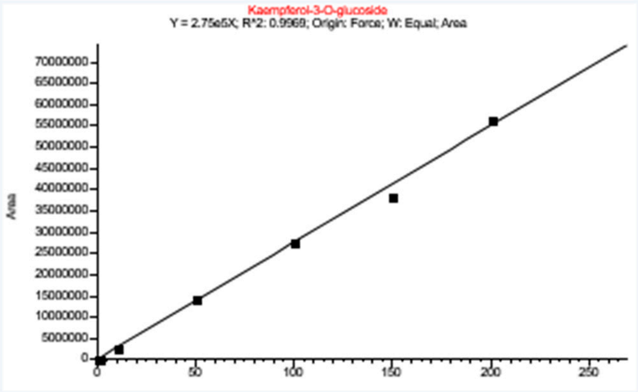
Kaempferol-3-O-glucoside
Y = 2.75e5X; R^2: 0.9969; Origin: Force; W: Equal; Area
Area

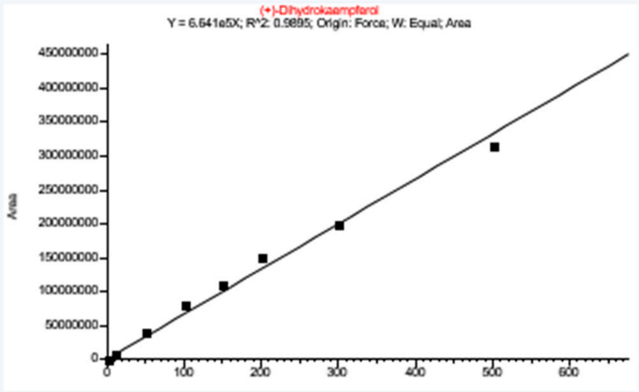
(+)-Dihydrokaempferol
Y = 6.641e5X; R^2: 0.9895; Origin: Force; W: Equal; Area
Area

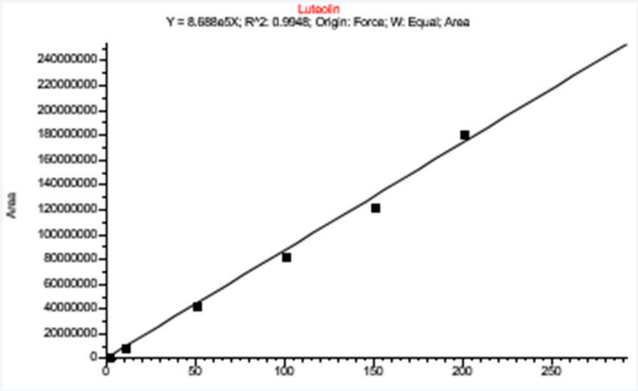
Luteolin
Y = 8.688e5X; R^2: 0.9948; Origin: Force; W: Equal; Area
Area

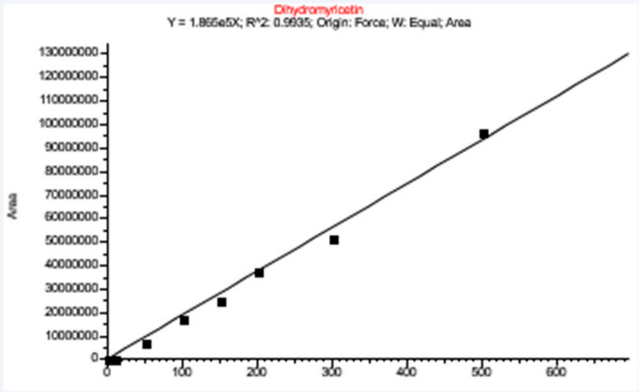
Dihydromyricetin
Y = 1.865e5X; R^2: 0.9935; Origin: Force; W: Equal; Area
Area

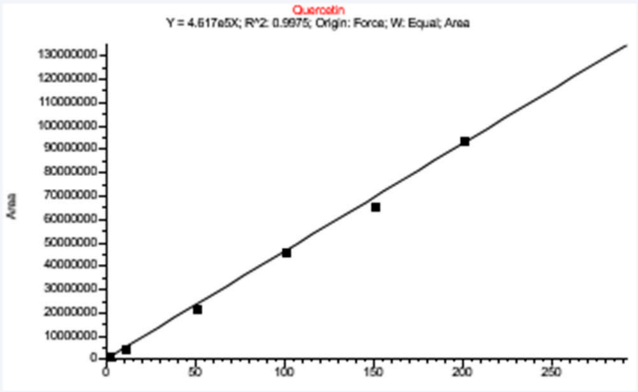
Quercetin
Y = 4.617e5X; R^2: 0.9975; Origin: Force; W: Equal; Area
Area

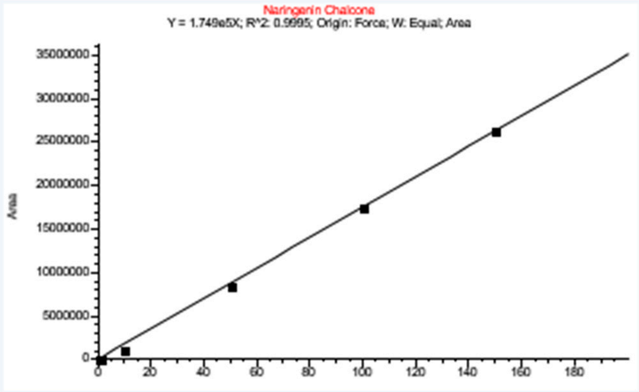
Naringenin Chalcone
Y = 1.749e5X; R^2: 0.9995; Origin: Force; W: Equal; Area
Area

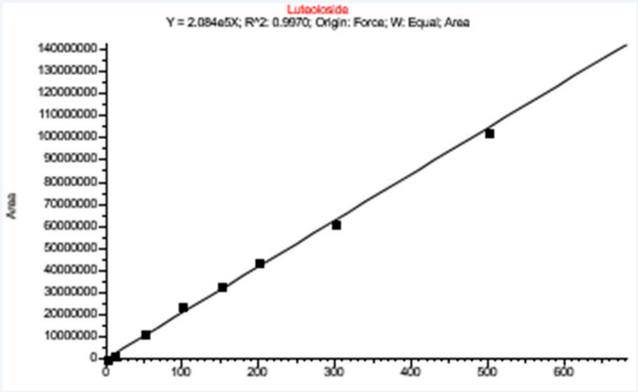
Luteoloside
Y = 2.084e5X; R^2: 0.9970; Origin: Force; W: Equal; Area
Area

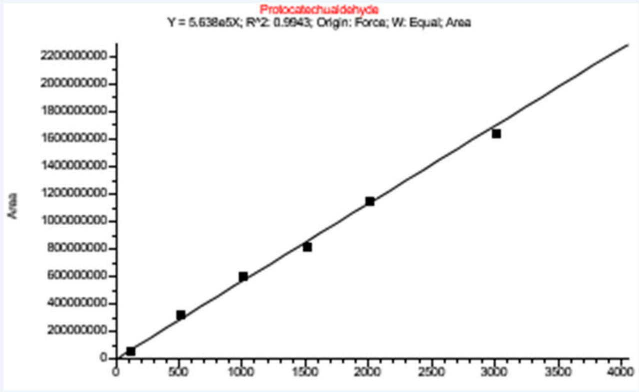
Protocatechualdehyde
Y = 5.638e5X; R^2: 0.9943; Origin: Force; W: Equal; Area
Area

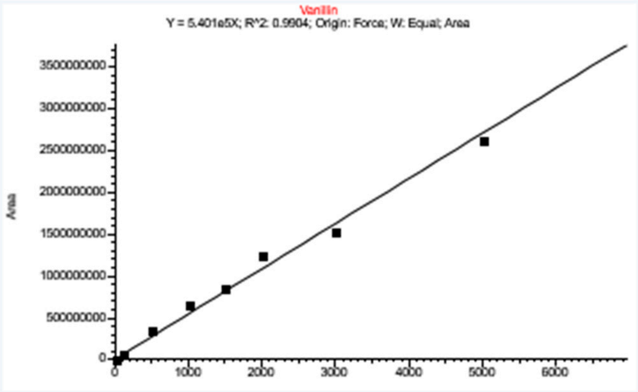
Vanillin
Y = 5.401e5X; R^2: 0.9904; Origin: Force; W: Equal; Area
Area

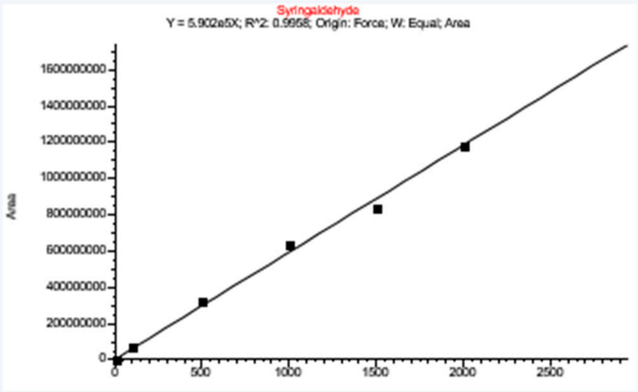
Syringaldehyde
Y = 5.902e5X; R^2: 0.9958; Origin: Force; W: Equal; Area
Area

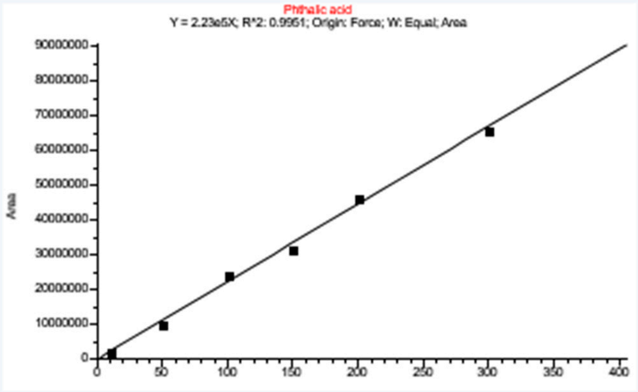
Phthalic acid
Y = 2.23e5X; R^2: 0.9951; Origin: Force; W: Equal; Area
Area

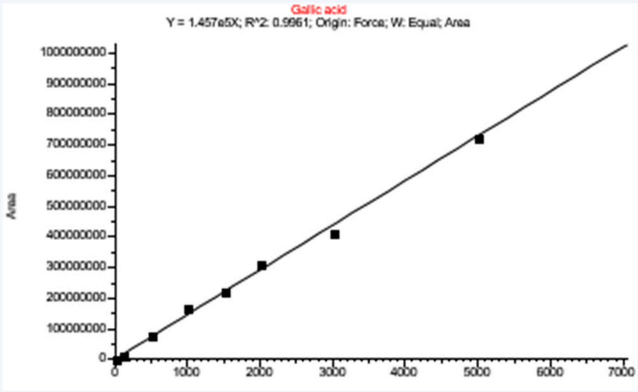
Gallic acid
Y = 1.457e5X; R^2: 0.9961; Origin: Force; W: Equal; Area
Area

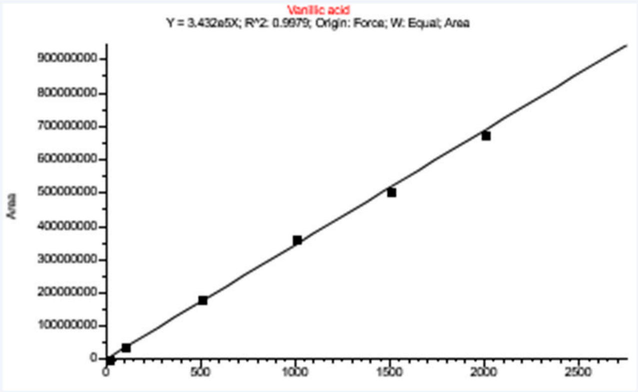
Vanillic acid
Y = 3.432e5X; R^2: 0.9979; Origin: Force; W: Equal; Area
Area

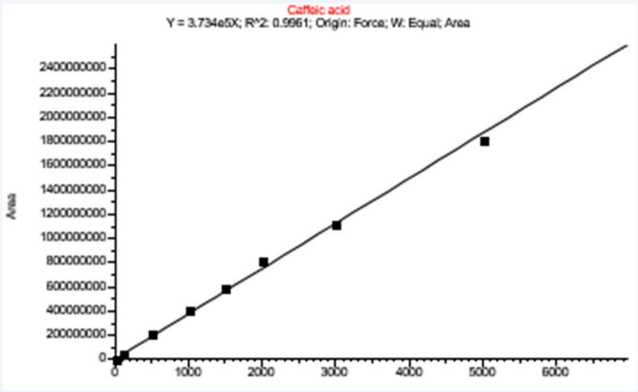
Caffeic acid
Y = 3.734e5X; R^2: 0.9961; Origin: Force; W: Equal; Area
Area

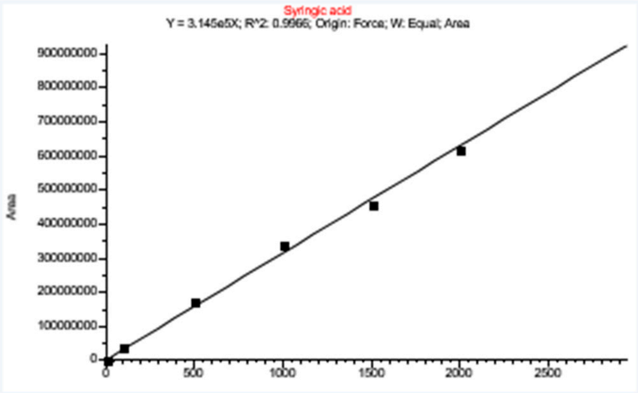
Syringic acid
Y = 3.145e5X; R^2: 0.9966; Origin: Force; W: Equal; Area
Area

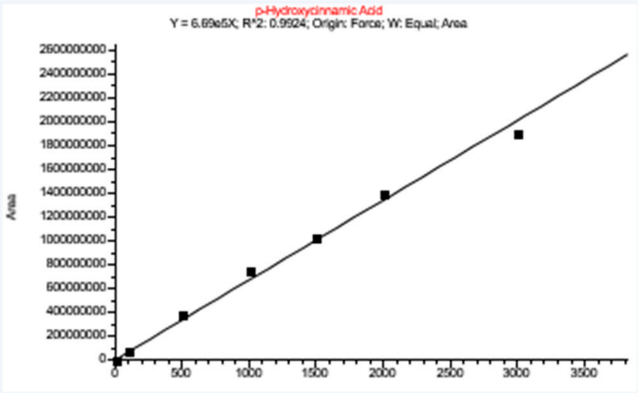
p-Hydroxycinnamic Acid
Y = 6.69e5X; R^2: 0.9924; Origin: Force; W: Equal; Area
Area

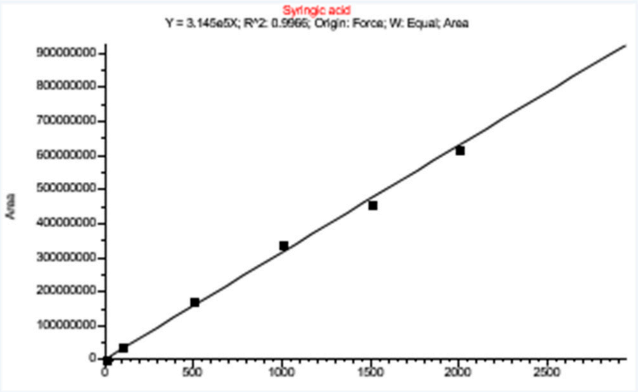
Syringic acid
Y = 3.145e5X; R^2: 0.9966; Origin: Force; W: Equal; Area
Area
900000000
800000000
700000000
600000000
500000000
400000000
300000000
200000000
100000000
0
0
500
1000
1500
2000
2500

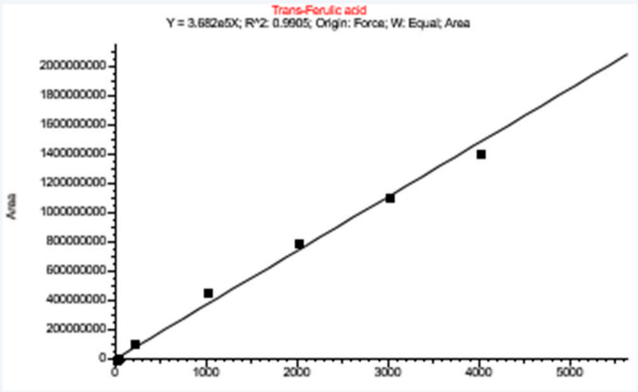
Trans-Ferulic acid
Y = 3.682e5X; R^2: 0.9905; Origin: Force; W: Equal; Area
Area
2000000000
1800000000
1600000000
1400000000
1200000000
1000000000
800000000
600000000
400000000
200000000
0
0
1000
2000
3000
4000
5000

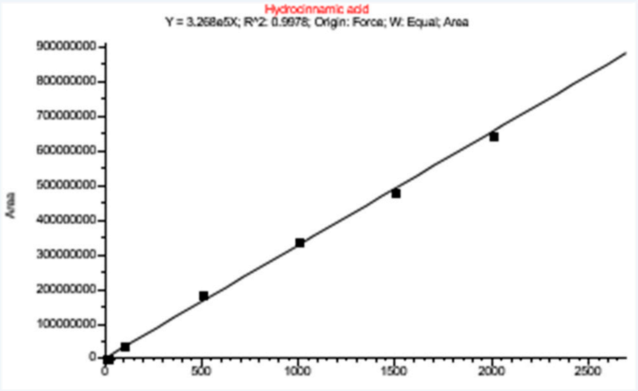
Hydrocinnamic acid
Y = 3.268e5X; R^2: 0.9978; Origin: Force; W: Equal; Area
Area
900000000
800000000
700000000
600000000
500000000
400000000
300000000
200000000
100000000
0
0
500
1000
1500
2000
2500

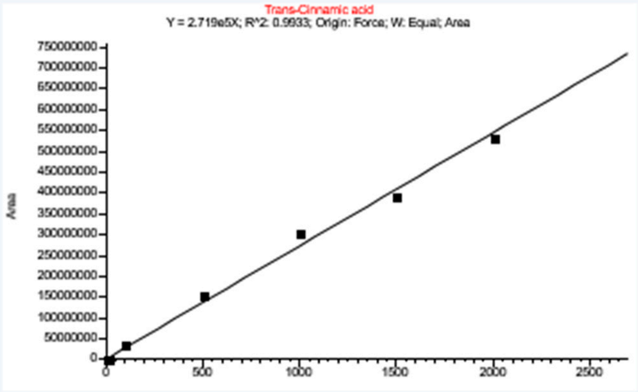
Trans-Cinnamic acid
Y = 2.719e5X; R^2: 0.9933; Origin: Force; W: Equal; Area
Area
750000000
700000000
650000000
600000000
550000000
500000000
450000000
400000000
350000000
300000000
250000000
200000000
150000000
100000000
50000000
0
0
500
1000
1500
2000
2500
